# Genetic modifiers of cancer risk in Lynch syndrome: a review

**DOI:** 10.1007/s10689-013-9614-2

**Published:** 2013-03-08

**Authors:** Bente A. Talseth-Palmer, Juul T. Wijnen, Desma M. Grice, Rodney J. Scott

**Affiliations:** 1 School of Biomedical Sciences and Pharmacy, University of Newcastle, Newcastle, Australia; 2 Hunter Medical Research Institute, Newcastle, Australia; 3 Centre for Human and Clinical Genetics, Leiden University Medical Centre, Leiden, The Netherlands; 4 Preventative Health Flagship, CSIRO, North Ryde, NSW 2113 Australia; 5 Animal, Food and Health Sciences, CSIRO, North Ryde, NSW 2113 Australia; 6 Division of Molecular Medicine, Hunter Area Pathology Service, John Hunter Hospital, Hunter New England Health District, Lookout Road, New Lambton, NSW 2305 Australia

**Keywords:** Lynch syndrome, Modifier genes, Disease expression, CRC susceptibility loci

## Abstract

The report by Aldred Scott Warthin in 1913 of a cancer family history and expanded on by Henry T. Lynch demonstrated one of the most enduring traits observed in patients with Lynch syndrome. The recognition of a variety of malignancies occurring at differing ages within a single family suggested the role of genetic variance on disease expression in an autosomal dominantly inherited genetic condition. With the identification of the genetic basis of Lynch syndrome and the subsequent collection of families and their medical records it has become possible to identify subtle genetic effects that influence the age at which disease onset occurs in this cancer predisposition. Knowledge about genetic modifiers influencing disease expression has the potential to be used to personalise prophylactic screening measures to maximise the benefits for family members and their carers.

## Introduction

The primary function of mismatch repair (MMR) genes is to eliminate base–base mismatches and insertion-deletion loops which arise as a consequence of DNA polymerase slippage during DNA replication [1]. MMR confers several genetic stabilisation functions; it corrects DNA biosynthesis errors, ensures the fidelity of genetic recombination and participates in the earliest steps of checkpoint and apoptotic responses [2, 3].

Lynch syndrome (LS) is associated with a breakdown in the efficiency of DNA MMR as a result of the loss of one or more DNA repair proteins from this process. Mutations in *MSH2, MLH1, MSH6* or *PMS2* decrease the fidelity of DNA replication as there is a failure to recognise and replace errors resulting from the mis-incorporation of bases by DNA polymerase. DNA MMR is a housekeeping function of all nucleated cells and as such any breakdown in the fidelity of this process is likely to result in disease irrespective of which gene is affected. Furthermore, mutations in DNA MMR genes result in a “mutator phenotype” thereby predisposing individuals to a significantly increased risk of malignancy.

It has been obvious from the first *MSH2* and *MLH1* mutation reports that differences in the ages of cancer diagnosis in patients harbouring germline mutations in DNA MMR genes do occur both within and between families. Furthermore, unrelated families harbouring the same mutation present with different disease profiles [4–6].

The differences in disease expression both within and between families harbouring the same mutation are most likely a result of environmental, genetic or a mixture of both influences. The search for environmental factors that could account for phenotypic variation is an almost intractable problem when studied retrospectively and is best undertaken prospectively, where as many factors can be included in any such analysis. In contrast, genetic factors can be studied retrospectively and therefore are more amenable to investigation. Ideally, both genetic and environmental factors should be studied together to identify those factors that can be modified by appropriate intervention. To the authors knowledge no such study has been undertaken to date and only genetic modifiers have been identified in LS at this time.

## Modifier genes

The search for modifier genes has been ongoing ever since the first LS families were identified. Initial studies focused on genes associated with xenobiotic metabolism which have been followed by genes involved in the immune response, DNA repair, cell cycle control and as yet undefined genomic regions identified as a result of large genome wide association studies searching for genetic risk factors for colorectal cancer.

Initial studies in the search for genetic modifiers of disease focused on the biological plausibility of functional variants in a variety of different pathways that include but were not limited to xenobiotic clearance [7–12], cell cycle control, DNA repair [13–16], immunological activity and glycolysis [17–19]. The first reports on modifier genes also tended to be from small cohorts of patients [7–11, 13] where the confidence intervals of association were large. Many of the original studies have subsequently failed replication in larger cohorts, suggesting that the population size used in many of the initial reports was too small and therefore lacked statistical rigour. Nevertheless, some inferences were proposed that have appeared to hold up to greater scrutiny.

## Xenobiotic clearance and micronutrient metabolism

The removal of many carcinogens is controlled by a complex process involving phase I enzymes such as cytochrome P450 (CYP), and phase II enzymes that include the glutathione-S-transferases (GSTs) and N-acetyl transferases (NATs) [20]. Polymorphisms in these genes have been associated with colorectal cancer but the precise roles that each variant has on cancer risk remains controversial [10–12, 21–28]. Genes involved in xenobiotic metabolism were therefore considered as ideal candidate modifier genes, as a result of their association with the risk of malignancy [20]. In the context of LS only a few reports have been forthcoming on the disease modifying effects of xenobiotic enzymes and have focused on *NAT1*, *NAT2*, *GST* and *CYP*.

The first study of an association between disease risk and polymorphisms in *NAT2* in LS patients was reported in a small number of families where there was considerable disease diversity [7] and was later replicated in a second independent report [11]. Re-investigation of the association in two other unrelated patient cohorts failed to confirm any association [12, 24]. The failure to identify an association with NAT2 has since been indirectly confirmed in a review by Brockton et al. 2000 [28] who showed in 10 out of 11 studies of invasive CRC that *NAT2* genotypes were not linked to disease risk.

Polymorphisms in *GST* and cytochrome p450 family 1, subfamily A, polypeptide 1 (*CYP1A1*) genes and their relationship to disease risk in LS have also been reported and have since come under scrutiny. There have been reports both for and against an association [8, 10, 12, 29]. In one study the Msp1 wildtype allele of *CYP1A1* was associated with a decreased risk of CRC but the allele distribution was not in Hardy–Weinberg equilibrium [12] thereby casting some doubt on the relationship. In this instance the evidence suggests that either a genotyping error that skews the results in favour of an association that is not real or it can be taken as supporting evidence for a correlation [30]. Two polymorphisms in *CYP1A1* have been associated with CRC [25, 27], which taken together with the report of Talseth et al. 2006 [12] suggests that this gene is involved in some aspect of CRC development.

The association of xenobiotic clearance and disease expression in LS is complex and likely to be heavily influenced by environmental factors that are not easily identified or quantified. Nevertheless, future studies should take into consideration gene environment interactions to fully explain the contribution of xenobiotic enzyme polymorphisms with disease risk. This is highlighted in the findings from European compared to Asian populations where polymorphisms in GST show an association in the Korean population but not Australian or European [7, 10, 29].

Taken together, the assessment of xenobiotic modifier genes requires additional studies to delineate the environmental factors that in concert with their respective genetic variation affect the risk of disease.

## Cell cycle control

Since DNA repair is integrally associated with cell cycle control, functional polymorphisms in genes associated with this aspect of genomic integrity are attractive candidates for modifier gene studies. The most well studied gene in this regard is the tumour suppressor gene *TP53*. *TP53* is the most frequently mutated gene in a variety of cancers that include colorectal cancer [31]. *TP53* has been called a master regulator as it is involved in the maintenance of genomic integrity, blocking cell proliferation after DNA damage and initiating apoptosis if it is too extensive [32, 33]. In addition, there exists within any given population a common functional variant, R72P, which is estimated to occur at a frequency of approximately 35 % in the general population [34]. The R72P SNP alters the function of TP53 [35, 36] and as such has been widely studied in a variety of malignancies [13, 37, 38].

In 2004 the age of colorectal cancer diagnosis in LS patients was found to be associated with the R72P polymorphism [13]. Subsequently, this association could not be replicated [15, 39]. The failure to identify an association with *TP53* suggested that the positive effect observed in the first study [13] may have been related to the TP53 partner MDM2 that is also polymorphic. The effect of the polymorphism is to increase levels of MDM2 that results in the inability to stabilise TP53’s cellular stress response [40]. Evidence implicating *MDM2* as a modifier gene could not be found in other studies [16].

*Aurora*-*A* and *Cyclin D1*, both necessary for cell cycle control, have also been associated with the age of onset of CRC in LS patients [41, 42] but replication studies have consistently failed to substantiate the initial findings [43]. Several reports in particular have focused on *Cyclin D1* and most demonstrate no association [42, 44, 45]. In an Australian and Polish study an initial report suggested an association with *MSH2* mutation carriers [43] however, on expansion of the study population the original effect disappeared [46].

*ATM* is another potential modifier that is involved in the control of the cell cycle. Two reports [14, 47] have examined a variant within the ATM gene producing diametrically opposed results. At this time, no conclusions can be made with respect to the potential role of *ATM* as a modifier gene in LS.

## DNA repair

The role of DNA repair processes outside of the context of DNA mismatch repair represents a salient mechanism that could influence the age at which disease develops in LS. There are over 130 genes involved in DNA repair that all have significant roles in maintaining the veracity of the genome [48]. The DNA repair pathways of MMR and base excision repair (BER) are both involved in the identification, removal and repair of replication induced DNA errors. The MMR system involves correcting mismatched bases that occur during DNA replication [1], whereas BER is highly specific for the repair of oxidative DNA damage [49]. Double-strand breaks (DSBs) in DNA are repaired by either non-homologous end joining (NHEJ) or homologous recombination (HR). Polymorphisms in DNA repair genes have been associated with cancer susceptibility suggesting that altered repair function may explain some of the phenotypic differences observed in LS. Only one report to date has examined a series of DNA repair gene polymorphisms in *MSH3*, *OGG1, XRCC1, XRCC2, XRCC3, BRCA2* and *Lig4* to determine if any could be associated with disease expression in LS [50]. None of the polymorphisms in the DNA repair genes listed could be shown to influence disease risk. The failure to identify any modifying effect does not rule out the possibility that there exist DNA repair gene polymorphisms that influence disease risk. Further studies of additional genes are required before it can be unequivocally stated that DNA repair gene polymorphisms are not associated with disease expression.

Telomerase is an enzyme involved in maintaining telomere length after cell division. Telomere shortening has been linked to the initiation of epithelial malignancies and chromosomal instability [51, 52]. A polymorphism in *hTERT* has been associated with cancer risk and one report has tentatively linked this polymorphism to an earlier age of cancer and/or polyp development in patients with LS [53]. Of interest in this report is the absence of effect in patients older than 45 years of age, suggesting that this modifier is no longer effective when telomere shortening has occurred in aging populations [54].

## Immunological function

Tumours development is enhanced by an environment that supports tumour growth by promoting angiogenesis and facilitating genomic instability. The quintessential example is Crohns’ disease where an increased risk of developing CRC is observed if the disease is left untreated [55]. Crohns’ disease is an auto-immune disorder characterised by an over active pro-inflammatory response [56, 57]. Inflammatory responses can also increase DNA damage, growth stimulation and enhanced survival of damaged cells [54, 56]. Many cytokines are polymorphic with effects that can alter transcription level and activity both in pro- and anti-inflammatory response genes.

Several polymorphisms in a number of cytokines have been investigated in relation to CRC risk and other cancer types but not for LS [58–65]. Genetic variation in pro- and anti-inflammatory cytokines has also been shown to influence the response to carcinogen exposure [64] [57] thereby suggesting that the immune response is integral to disease risk. With respect to LS no association has been identified in the one report focusing on a series of cytokine SNPs and disease expression [17].

Given the complexity of the inflammatory response and the limited number of SNPs examined, it cannot be ruled out that a relationship between SNPs influencing the immune response and LS exits.

## Growth factors

Many growth factors are functionally polymorphic [66, 67] and have been shown to be associated with a variety of malignancies [68–71]. One growth factor that has received some attention is IGF-1. Several environmental and physiological reasons have been proposed that influence IGF-1 expression; however it has been only recently that evidence has accumulated suggesting a genetic role. Rosen et al. was the first to report that the length of the CA repeat region in *IGF*-*1* may be associated with circulating IGF-1 levels [67].

In the context of LS IGF-1 appears to be particularly important, *IGF*-*1*. The function of *IGF*-*1* is associated with cellular proliferation and differentiation and elevated levels of IGF-1 have been linked to CRC which is thought to be a result of the mitogenic and anti-apoptotic effects elicited by this protein [70, 71]. *IGF*-*1* was first reported as a potential modifying gene in LS disease expression in 2006 [18]. The CA-repeat polymorphism located near the *IGF*-*1* promoter region was described as having an association with the age of disease onset in a cohort of 121 LS participants originating from the United States [18]. This result has been replicated in two additional populations, one from Australia [72] and a second from Poland [19]. Intriguingly, not only was there a relationship between CA repeat size but it appeared that the shorter CA-repeat the greater the effect. Given the paucity of replication of modifier gene effects it is encouraging to observe consistent effects are retained across different ethnicities [19].

The identification of a CA-repeat polymorphism in a growth factor gene associated with the age of colorectal cancer onset in LS suggests other CA-repeats that are functionally important in growth factor expression should be examined for their potential role as modifier genes in LS.

## Other modifiers

A series of other modifier genes have been identified that appear to influence disease expression in LS. These include but are not limited to methylene tetrahydrofolate reductase (MTHFR) gene, the gene associated with haemochromatosis (Hfe) and a variety of other polymorphisms that occur in regions of the genome that do not as yet have any defined function. The latter polymorphisms have been identified from genome wide association studies examining genetic risk factors associated with colorectal cancer in the general population. A brief summary of what has been revealed follows:

### Methylene tetrahydrofolate reductase (MTHFR)

There are a number of reports in the literature suggesting that polymorphisms in MTFHR are associated with colorectal cancer risk. Two functional polymorphisms in MTHFR (*C677T* and *A1298C*) have been the subject of intense scrutiny in relation to colorectal cancer risk as they both have significant effects on the activity of the protein product [73, 74]. These two polymorphisms occur in relatively high frequency in the general population both have been associated with altered enzymatic function. MTHFR is a key folate-metabolizing enzyme involved in DNA methylation and DNA synthesis. The enzyme catalyses the irreversible conversion of 5,10-methylenetetrahydrofolate (5,10-MTHF), needed for purine and thymidine synthesis, to 5-methyltetrahydrofolate (5-MTHF), which is necessary for methionine production. Insufficient thymidine results in uracil misincorporation into DNA, leading to single-strand and double-strand breaks. Any change in the relative frequency of DNA damage will increase the risk of genetic instability.

Both *A1298C* and *C677T* are in high linkage disequilibrium [74] and only rarely has a MTHFR allele been identified that carries both the homozygote (*C1298C/T677T*) variants of these polymorphisms [75–77]. Owing to this linkage disequilibrium, no studies have been reported where patients have inherited both homozygote variants in *cis*. Nevertheless, compound heterozygote carriers of 1298C and 677T have been identified. The effect of inheriting both alleles in *trans* effectively reduces overall MTHFR activity, thereby significantly altering the kinetics of folate metabolism. Evidence of the effects of MTHFR variants on disease expression in LS revealed that compound heterozygotes appeared to be significantly protected against an early age of disease onset [78]. The survival estimates predicted a median 10 year age difference for CRC onset in patients carrying the combined heterozygote MTHFR genotype which was supported by multi-variable regression modelling. The data also suggested this effect was significant in both *hMLH1* and *hMSH2* carriers, where previously only a significant association had been described in *hMLH1* for *C677T* only [79].

For individuals with a MMR deficiency, the effect of reduced MTHFR activity is potentially advantageous since uracil misincorporation could be particularly deleterious in conjunction with an impaired DNA repair pathway. Knowledge about the kinetics of MTHFR is significant in so far as dietary supplementation with folate (or withdrawal) may be a mechanism by which disease expression may be modulated in LS and may prove to be an indicator of individual disease risk in this syndrome.

### Haemochromatosis (Hfe)

The iron overload disorder hereditary haemochromatosis (HH) is characterised by high iron indices and progressive parenchymal iron overload due to unrestricted iron uptake [reviewed in [80–83]. The primary cause of classical HH is a result of polymorphisms in *HFE*, especially *845G* > *A* SNP which results in the substitution of a tyrosine residue for a cysteine at position 282 (C282Y), which is present in 10-15 % of individuals of northern European descent. A second more common but less penetrant polymorphism, *163C* > *G* SNP (H63D) is present in 15-30 % of individuals [80, 83–89]. Patients homozygote for the C282Y polymorphism are about 3 times more likely to develop CRC compared to matched controls without the mutation [97]. There has been only one study examining the risk of CRC in LS suggestive of an effect. Homozygosity of the *HFE* H63D mutation may act as a disease risk modifier in LS [90], with as much as a 6 year difference in the age of disease onset for this less penetrant HH allele. In the study by Shi et al.; [90] there were too few C282Y homozygotes to allow for any meaningful interpretation. While these findings will require substantiation in other populations, they support a possible relationship between iron dysregulation and colorectal cancer risk. An in-depth study of compound heterozygotes for both Hfe polymorphisms is required to firmly establish if iron status is indeed a risk factor for CRC in LS. It is well recognized that gender affects are significant in HH and males tend to fair less well than females. This may well be the case in LS as well but larger studies are necessary to assess the exact relationship of Hfe polymorphisms and colorectal cancer risk.

### DNA (cytosine-5-)-methyltransferase 3 beta (DNMT3B)

DNA methylation is regulated by a family of DNA methyltransferases (DNMTs), of which three active forms (DNMT1, DNMT3A and DNMT3B) have been identified in mammalian cells [91]. A polymorphism located within *DNMT3B* has been reported to influence enzyme expression as a result of altering its promoter activity. *DNMT3B* was proposed as a candidate in disease modifier due to its role in methylation. An example is the delta *DNMT3B* SNP, which was reported to be associated with an earlier age of CRC inset in LS [92]. A replication study, with over 400 individuals, failed to support the original report of an association between the age of CRC onset and the *DNMT3B* polymorphism [93]. The failure to confirm the potential modifying influence of a polymorphism in one population compared to another could be simply due to insufficient numbers of test subjects. If a polymorphism is an affect modifier its response should be similar no matter what population is examined even though it may not reach statistical significance. In the case of the delta *DNMT3B* SNP no such trend was observed suggesting that the original observation may not have been statistically rigorous.

### Polymorphisms identified from colorectal cancer genome-wide associations studies

There are several loci identified within the human genome that have been linked to CRC risk in the general population [94–99]. Many of the loci represent novel regions within the genome where little, if any, information is available concerning functional aspects of what these represent. Several groups have examined some of these SNPs in the context of their modifier effects. In 2009 two of the SNPs (rs16892766 and rs3802842) located on chromosomes 8q23.3 and 11q23.1, respectively, were shown to be associated with an increased risk of developing CRC in Dutch LS patients [100]. This result was partially confirmed in a combined Australian and Polish report, where instead of there being a generalised effect on all LS patients, only those with *MLH1* mutations were found to have an increased risk of CRC [101]. A third report from France, however, failed to replicate these findings [102]. More recently, a combined analysis of the Australian, Dutch and Polish LS totalling more than 1300 patients has confirmed the original findings and allowed for an additive analysis to determine whether one or more modifier alleles contribute further to disease risk [103]. At this point in time it is not entirely clear as to what functional effects rs3802842 has on disease risk as it resides in a region of chromosome 11 that harbours four open reading frames and does not result in any amino acid coding change thereby suggesting it may be regulatory in nature [104]. The SNP located on chromosome 8q23.3 maps to *UTP23* [104] where it is presumed to alter the functional activity of the encoded protein.

## Continuing the search for modifier genes

Thus far there is now some evidence to suggest that disease expression in LS is modified by genetic factors that are inherited independently of a causative mutation in one of the DNA mismatch repair genes. To date only a candidate gene (or locus) study has been performed to identify potential modifier genes in LS.

An alternative approach to screening candidate genes would be to undertake a genome-wide association study similar to that performed for carriers of *BRCA1* mutations which revealed a modifier locus on chromosome 19 [105]. This study required a total of 2383 *BRCA1* mutation carriers for the discovery phase of the project and a further 5986 *BRCA1* mutation carriers for the replication phase [105]. Given that the carrier frequency of *BRCA1* is greater than that of all MMR gene mutation carriers combined it remains challenging to accumulate sufficient numbers of LS patients for a genome-wide association study, especially when there is some evidence to suggest that modifier effects may be specific to each MMR gene subgroup.

## Summary

The search of modifier genes that influence disease expression in LS has revealed a number of potential candidates that could be used for individualised patient care. Several of the modifier genes reported to date are potentially valuable in terms of intervention strategies. Both Hfe and MTHFR must be confirmed in larger patient cohorts and if shown to be unequivocally associated with disease risks do offer avenues of potential risk reduction. Other candidate modifier loci do appear to be very promising as valuable additions to genetic screening for fine tuning surveillance strategies to maximise patient care and minimise unnecessary intervention. By including modifier genes/loci in risk algorithms it should be possible to tailor surveillance options for individual patients, which should allow for better outcomes in terms of patient acceptance of screening procedures resulting in reduced morbidity and mortality.
